# Supplementary material for: Correlations in sleeping patterns and circadian preference between spouses
Source: Commun Biol. 2023 Nov 13;6:1156. doi: 10.1038/s42003-023-05521-7 (PMC10643442; doi:10.1038/s42003-023-05521-7)
Supplement: Supplementary file 2 — Supplementary Information [file 42003_2023_5521_MOESM2_ESM.pdf]

Supplementary Note

Supplementary Note 1 - Sleep trait glossary

UK Biobank self-reported measures

Chronotype (diurnal preference) – Whether a person identifies as being a 'morning person' or an 'evening person' (ordered categorical variable of "definitely a morning person", "more of a morning than an evening person", "do not know", more of an evening than a morning person", and "definitely an evening person")

Ease of waking – Whether a person finds it easy to wake up in the morning (ordered categorical variable of "not at all easy", "not very easy", "fairly easy", "very easy")

Sleep duration – Average number of hours slept in 24 hours, including naps (continuous variable, hours)

Insomnia symptoms – Person has trouble falling asleep at night or wakes up in the middle of the night (ordered categorical variable of "never/rarely", "sometimes", and "usually")

Snoring – Whether a person reports that their partner or a close relative or friend complains about their snoring (binary variable of "yes" or "no")

UK Biobank accelerometer measures

L5-timing (activity timing) – Timing of the least active 5 hours of the day (continuous variable of hours elapsed since previous midnight). L5-timing represents activity later in the day (i.e. inversely related to morning preference chronotype).

Sleep duration – Average number of hours of sleep per night (continuous variable, hours)

Number of sleep episodes – Average number of nocturnal sleep episodes separated by at least 5 minutes of wakefulness per night (continuous variable, number of episodes)

Sleep efficiency - Sleep duration divided by time in bed (equivalent to time in bed) (continuous variable, proportion)

Supplementary Figures

Supplementary Figure 1 – Participant flow diagram

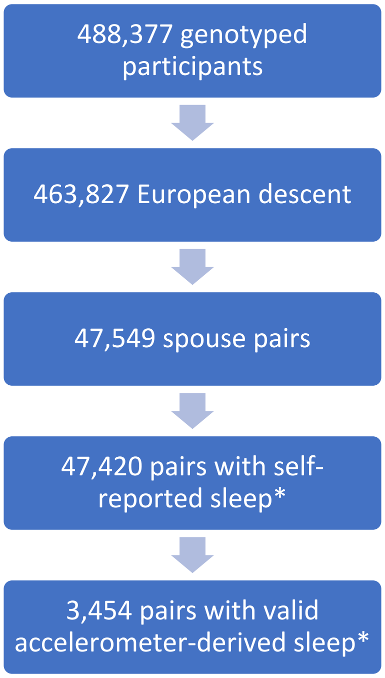

*At least one measure

Supplementary Figure 2 – Cross-trait spousal phenotypic correlations in UK Biobank

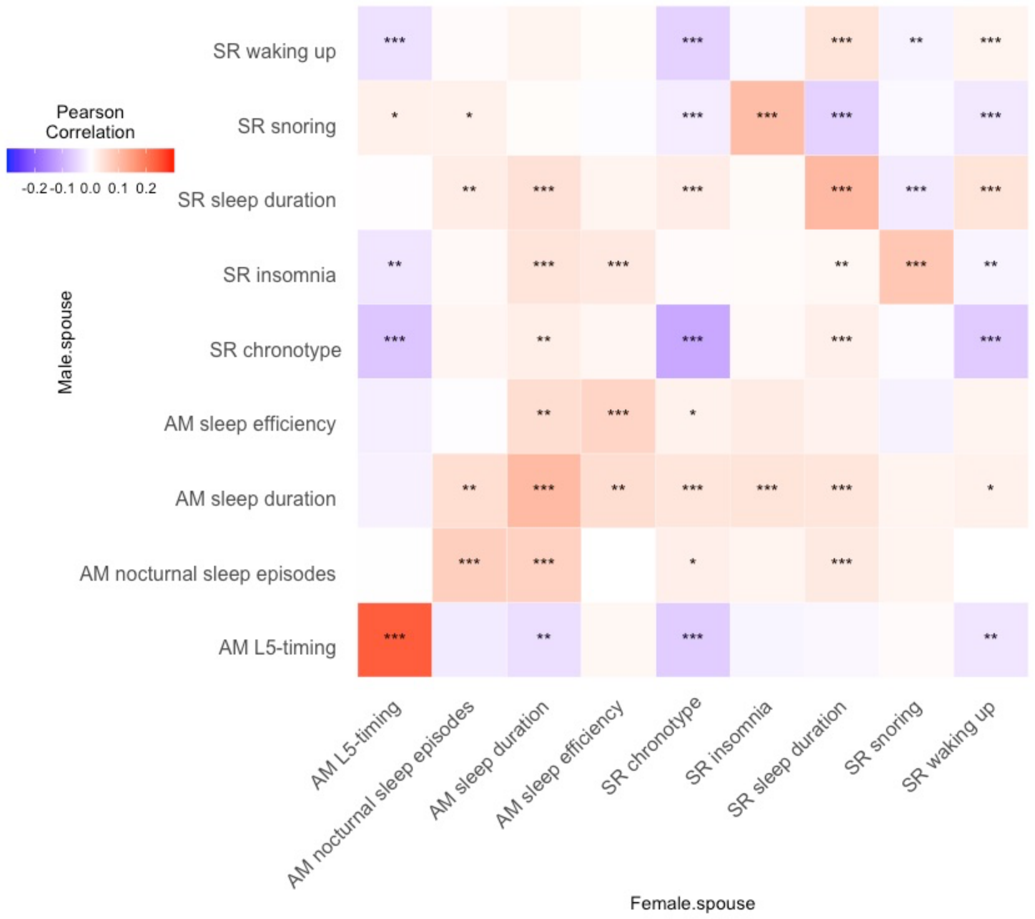

SR = self-reported; AM = accelerometer measured

*p<0.05, **p<0.01, ***p<0.001

Supplementary Figure 3 – Cross-trait spousal phenotypic correlations in 23andMe

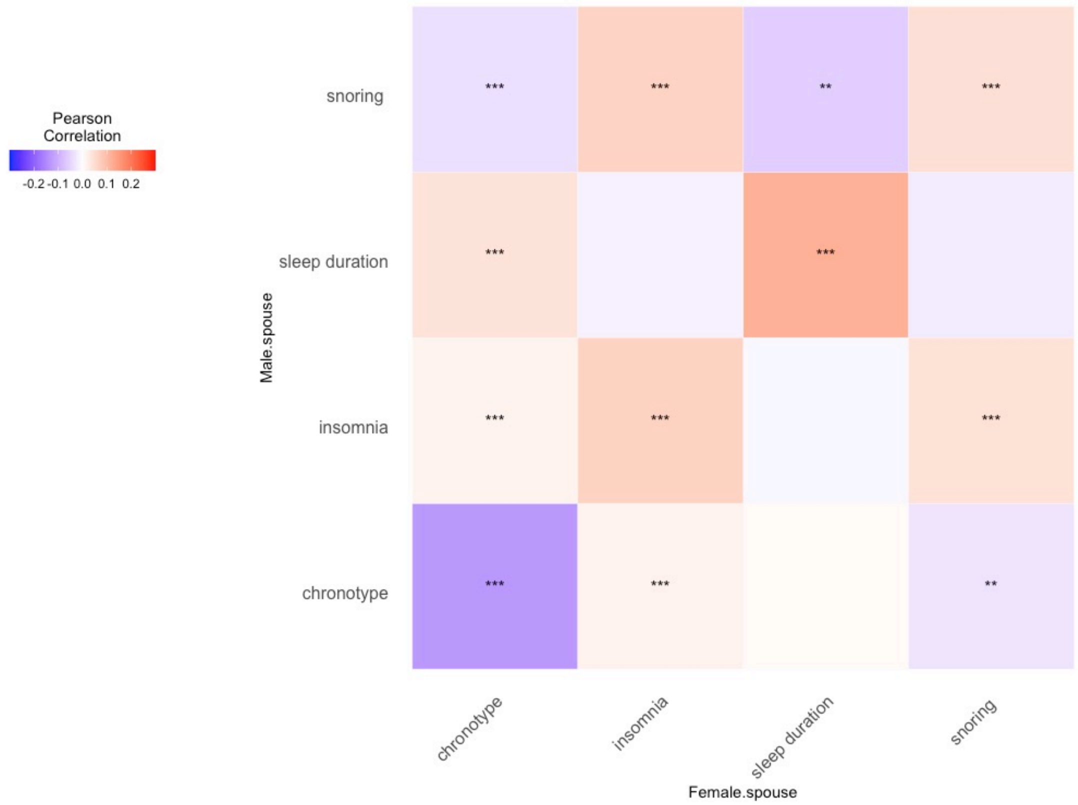

*p<0.05, **p<0.01, ***p<0.001

Supplementary Figure 4 - Comparison of causal estimates from Mendelian randomization between sexes in UK Biobank

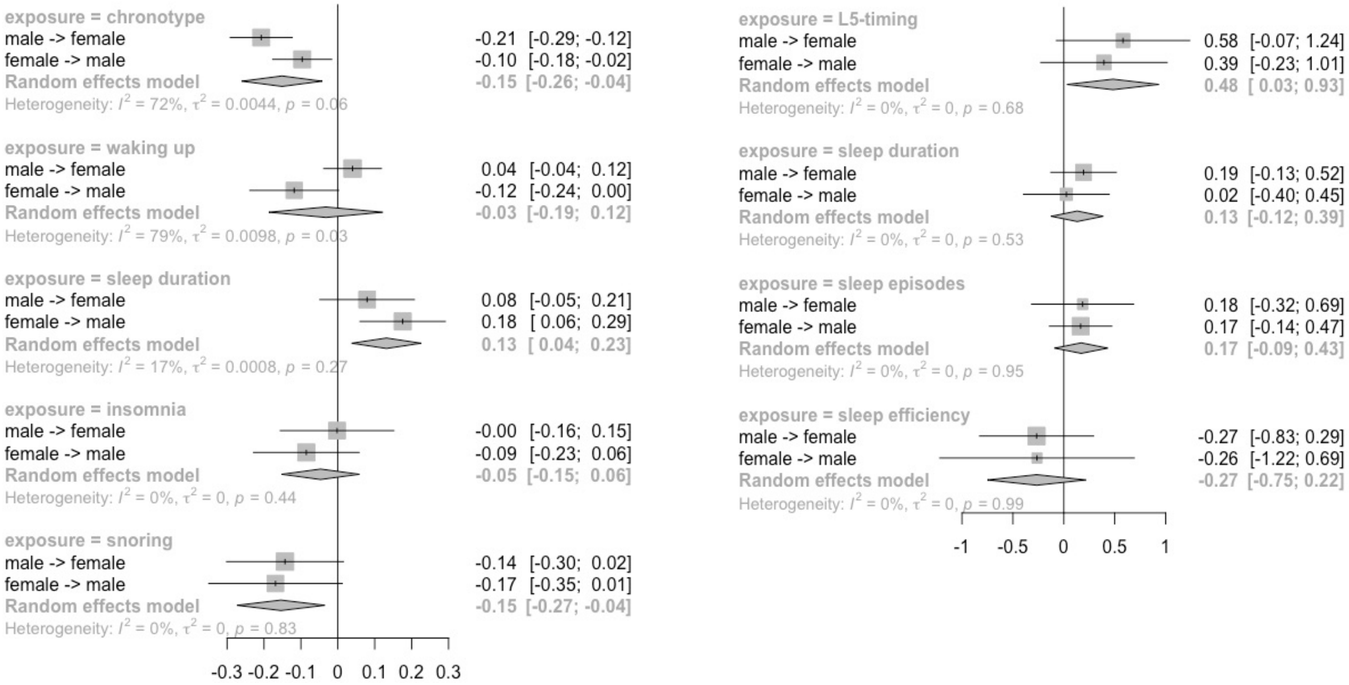

Effect estimates and accompanying 95% confidence intervals are shown.

Supplementary Figure 5 - Cross-trait causal estimates from Mendelian randomization in UK Biobank

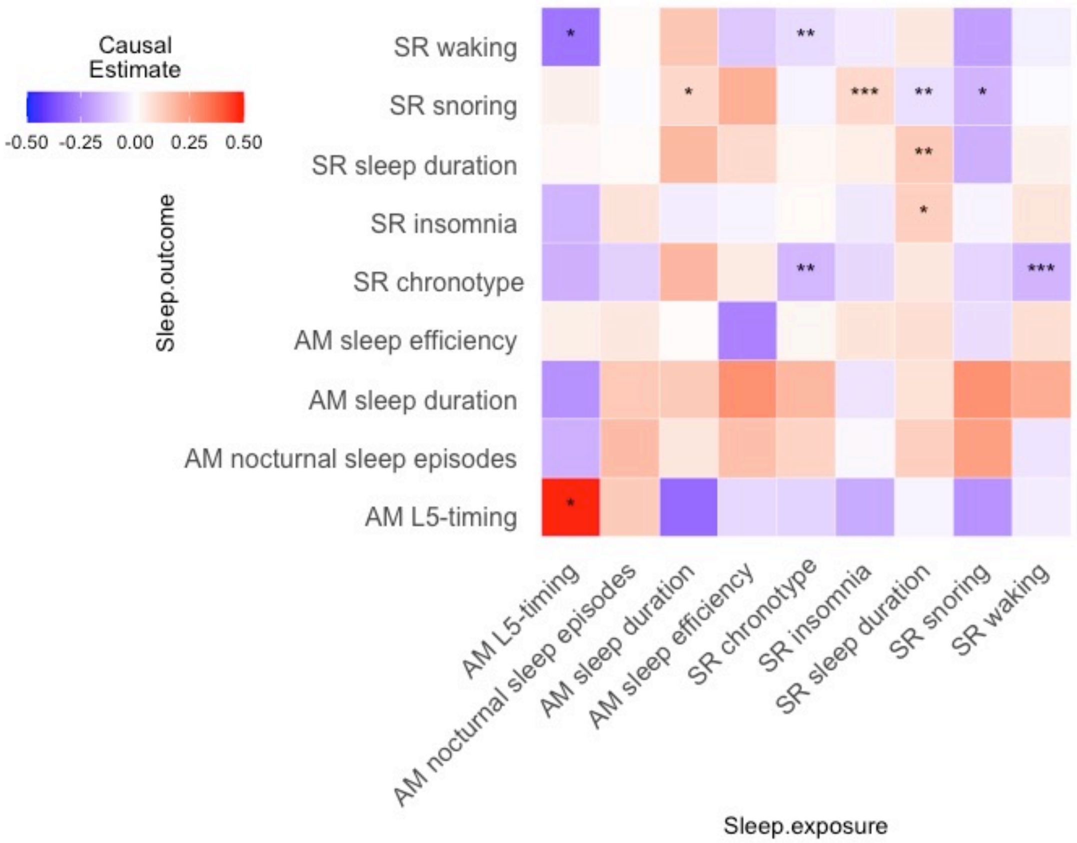

*p<0.05, **p<0.01, ***p<0.001

Supplementary Figure 6 - Spousal genotypic correlations between sleep traits in UK Biobank

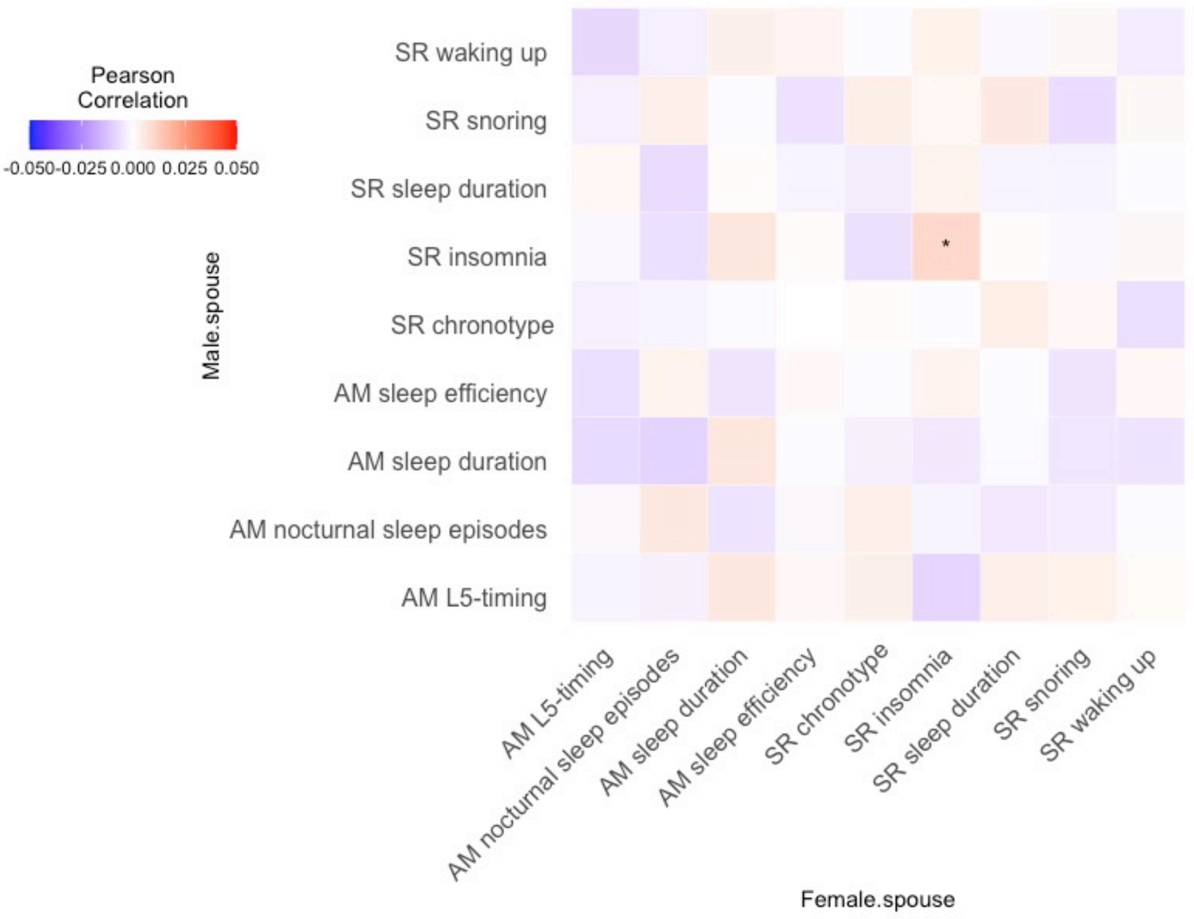

*p<0.05, **p<0.01, ***p<0.001

Supplementary Figure 7 – Genetic risk score correlations between spouses in UK Biobank

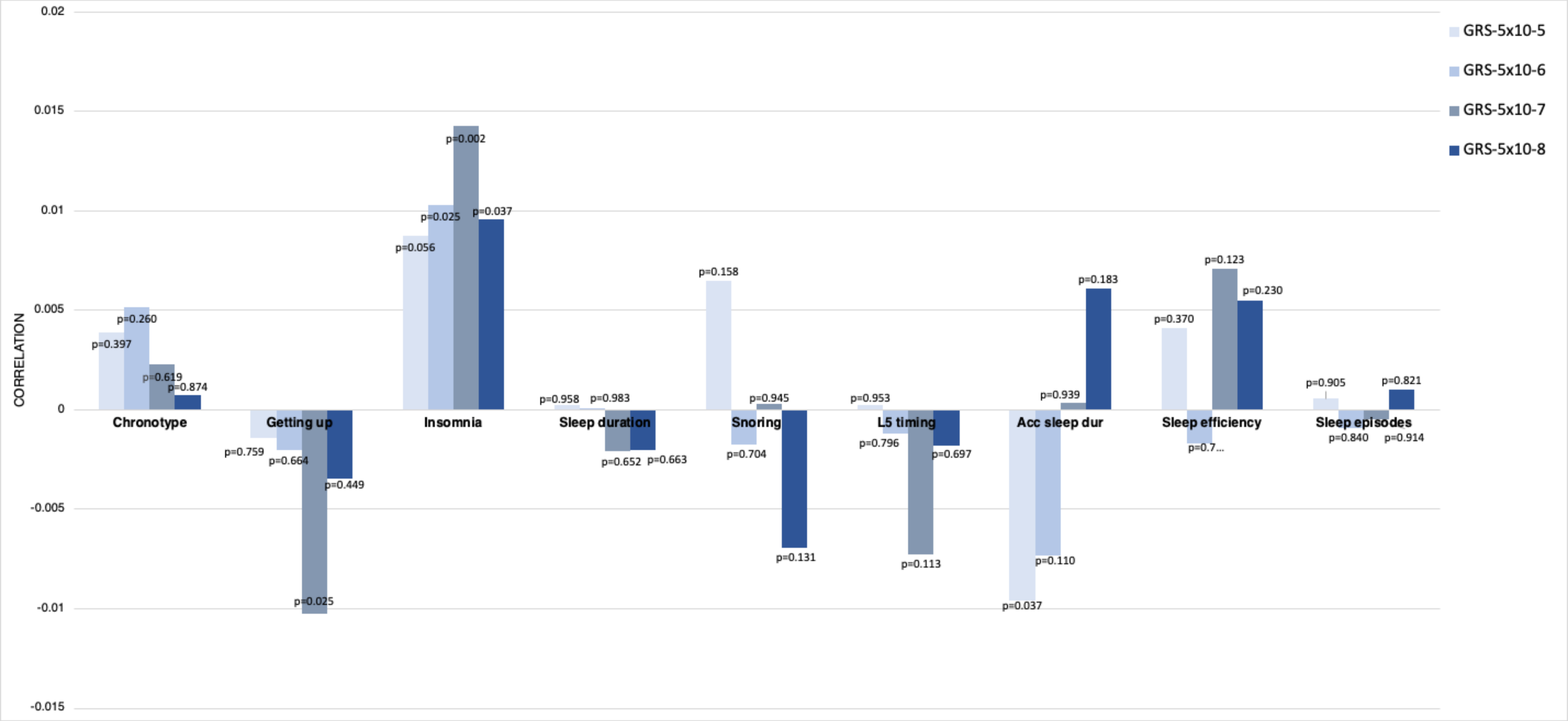

Supplementary Figure 8 – Effect modification by socio-economic, demographic and lifestyle factors and accelerometer characteristics

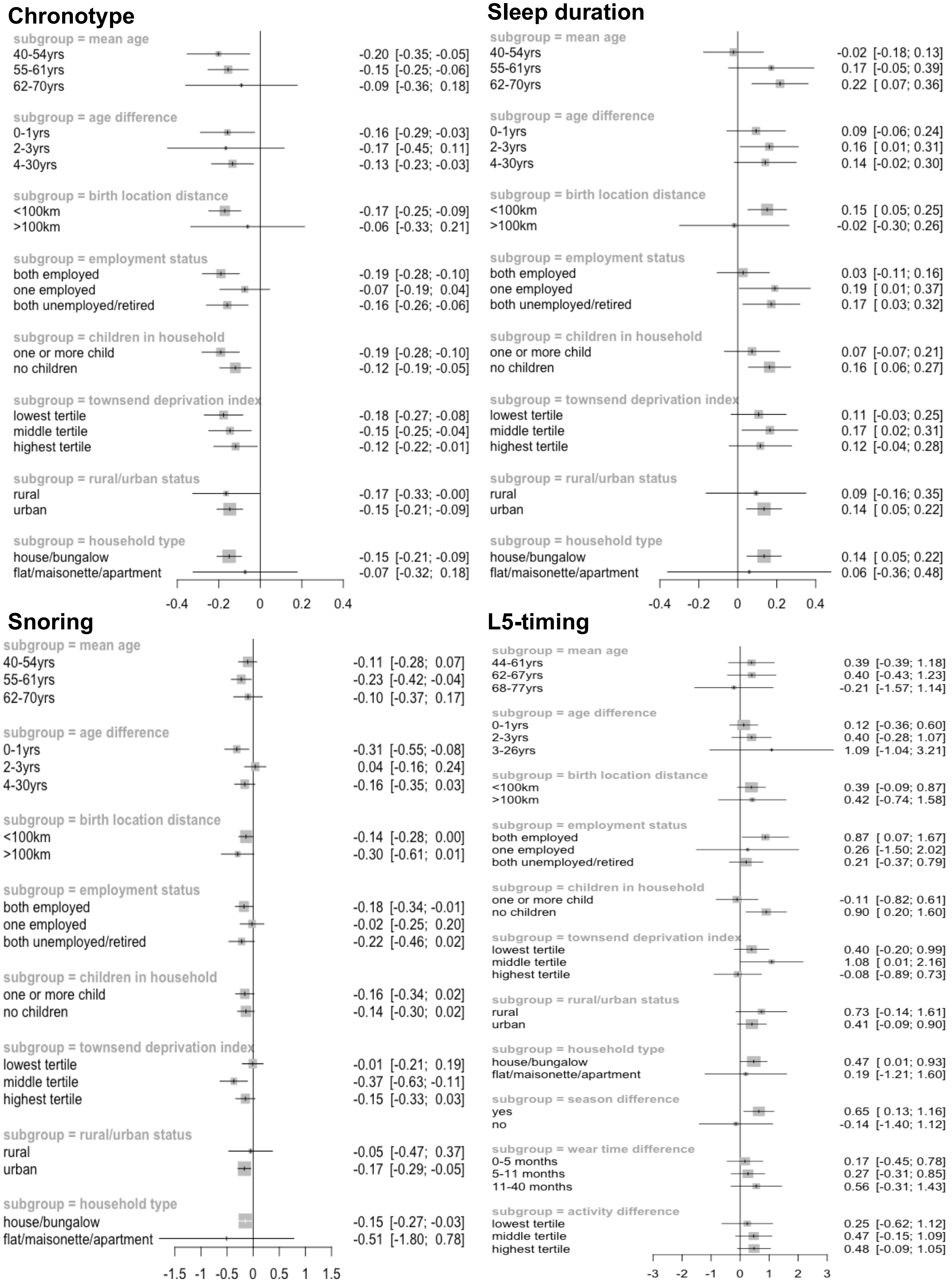

Effect estimates and accompanying 95% confidence intervals are shown.
