# Supplementary material for: Correlations in sleeping patterns and circadian preference between spouses
Source: Commun Biol. 2023 Nov 13;6:1156. doi: 10.1038/s42003-023-05521-7 (PMC10643442; doi:10.1038/s42003-023-05521-7)
Supplement: Supplementary file 3 — Description of Supplementary Files [file 42003_2023_5521_MOESM3_ESM.docx]

**Description of Additional Supplementary Files**

**File name:** Supplementary Data 1

**Description:** Table - Comparison of baseline characteristics between UK Biobank spouse pairs and other UK Biobank participants

**File name:** Supplementary Data 2

**Description:** Table - Comparison of baseline characteristics between UK Biobank spouse pairs with and without accelerometry data

**File name:** Supplementary Data 3

**Description:** Table - Sleep traits among individuals in different household categories in UK Biobank

**File name:** Supplementary Data 4

**Description:** Table - Sleep traits among male and female spouses in 23andMe

**File name:** Supplementary Data 5

**Description:** Table - Multivariable regression to assess associations between sleep traits among 23andMe spouse pairs

**File name:** Supplementary Data 6

**Description:** Table - Tests from pleiotropy-robust Mendelian randomization

**File name:** Supplementary Data 7

**Description:** Table - Pleiotropy-robust Mendelian randomization analyses of effects by sex

**File name:** Supplementary Data 8

**Description:** Table - Mendelian randomization using replicated SNPs to evaluate potential winner's curse

**File name:** Supplementary Data 9

**Description:** Table - Mendelian randomization using insomnia genetic risk score from Jansen et al.

**File name:** Supplementary Data 10

**Description:** Table - Genetic variants used in main Mendelian randomization analysis

**File name:** Supplementary Data 11

**Description:** Table - Genetic variants used in Mendelian randomization sensitivity analysis for Winner's curse

**File name:** Supplementary Data 12

**Description:** Table - Genetic variants associated with insomnia in Jansen et al. genome-wide association study

**File name:** Supplementary Data 13

**Description:** Source data for Figure 2

**File name:** Supplementary Data 14

**Description:** Source data for Figure 3

**File name:** Supplementary Data 15

**Description:** Source data for Figure 4
