# Supplementary material for: Correlations in sleeping patterns and circadian preference between spouses
Source: Commun Biol. 2023 Nov 13;6:1156. doi: 10.1038/s42003-023-05521-7 (PMC10643442; doi:10.1038/s42003-023-05521-7)
Supplement: Supplementary file 5 — Reporting summary [file 42003_2023_5521_MOESM5_ESM.pdf]

nature portfolio

Corresponding author(s): Rebecca Richmond
Celine Vetter

Last updated by author(s): Oct 21, 2023

Reporting Summary

Nature Portfolio wishes to improve the reproducibility of the work that we publish. This form provides structure for consistency and transparency in reporting. For further information on Nature Portfolio policies, see our Editorial Policies and the Editorial Policy Checklist.

Statistics

For all statistical analyses, confirm that the following items are present in the figure legend, table legend, main text, or Methods section.

n/a	Confirmed	
☐	☒	The exact sample size (*n*) for each experimental group/condition, given as a discrete number and unit of measurement
☐	☒	A statement on whether measurements were taken from distinct samples or whether the same sample was measured repeatedly
☐	☒	The statistical test(s) used AND whether they are one- or two-sided<br>*Only common tests should be described solely by name; describe more complex techniques in the Methods section.*
☐	☒	A description of all covariates tested
☐	☒	A description of any assumptions or corrections, such as tests of normality and adjustment for multiple comparisons
☐	☒	A full description of the statistical parameters including central tendency (e.g. means) or other basic estimates (e.g. regression coefficient) AND variation (e.g. standard deviation) or associated estimates of uncertainty (e.g. confidence intervals)
☐	☒	For null hypothesis testing, the test statistic (e.g. *F*, *t*, *r*) with confidence intervals, effect sizes, degrees of freedom and *P* value noted<br>*Give P values as exact values whenever suitable.*
☒	☐	For Bayesian analysis, information on the choice of priors and Markov chain Monte Carlo settings
☒	☐	For hierarchical and complex designs, identification of the appropriate level for tests and full reporting of outcomes
☐	☒	Estimates of effect sizes (e.g. Cohen's *d*, Pearson's *r*), indicating how they were calculated

Our web collection on statistics for biologists contains articles on many of the points above.

Software and code

Policy information about availability of computer code

Data collection	No software used.
Data analysis	"TwoSampleMR" package in R (version 3.5.1) ; "ivreg2" command in Stata (version 15).

For manuscripts utilizing custom algorithms or software that are central to the research but not yet described in published literature, software must be made available to editors and reviewers. We strongly encourage code deposition in a community repository (e.g. GitHub). See the Nature Portfolio guidelines for submitting code & software for further information.

Data

Policy information about availability of data

All manuscripts must include a data availability statement. This statement should provide the following information, where applicable:

- Accession codes, unique identifiers, or web links for publicly available datasets
- A description of any restrictions on data availability
- For clinical datasets or third party data, please ensure that the statement adheres to our policy

Summary-level data for UK Biobank and 23andMe are fully disclosed in the manuscript. Individual-level data are not publicly available due to participant confidentiality, and in accordance with the IRB-approved protocol under which the study was conducted. For details on accessing these studies, please contact access@ukbiobank.ac.uk and apply.research@23andme.com.

Field-specific reporting

Please select the one below that is the best fit for your research. If you are not sure, read the appropriate sections before making your selection.

☐ Life sciences ☒ Behavioural & social sciences ☐ Ecological, evolutionary & environmental sciences

For a reference copy of the document with all sections, see nature.com/documents/nr-reporting-summary-flat.pdf

Behavioural & social sciences study design

All studies must disclose on these points even when the disclosure is negative.

Study description	In this study, we aimed to investigate similarities in sleeping patterns between spouses in UK Biobank and 23andMe, Inc. (n=174,455 spouse-pairs).
Research sample	Results: Sleep characteristics between spouse-pairs ; Methods: UK Biobank and 23andMe
Sampling strategy	The present study uses data on 47,420 determined spouse-pairs within the UK Biobank to evaluate correlation in sleep traits between spouses, with replication in 23andMe (n≤127,035 spouse-pairs)
Data collection	Methods: UK Biobank and 23andMe
Timing	Methods: UK Biobank and 23andMe
Data exclusions	Supplementary Figure 1
Non-participation	Supplementary Figure 1
Randomization	N/A

Reporting for specific materials, systems and methods

We require information from authors about some types of materials, experimental systems and methods used in many studies. Here, indicate whether each material, system or method listed is relevant to your study. If you are not sure if a list item applies to your research, read the appropriate section before selecting a response.

Materials & experimental systems

n/a	Involved in the study	
☒	☐	Antibodies
☒	☐	Eukaryotic cell lines
☒	☐	Palaeontology and archaeology
☒	☐	Animals and other organisms
☐	☒	Human research participants
☒	☐	Clinical data
☒	☐	Dual use research of concern

Methods

n/a	Involved in the study	
☒	☐	ChIP-seq
☒	☐	Flow cytometry
☒	☐	MRI-based neuroimaging

Human research participants

Policy information about studies involving human research participants

Population characteristics	See above
Recruitment	Methods: UK Biobank and 23andMe
Ethics oversight	Methods: UK Biobank and 23andMe

Note that full information on the approval of the study protocol must also be provided in the manuscript.
